# Identifying person-level factors to guide digital mental health treatments for cancer survivors: an ecological momentary assessment study

**DOI:** 10.1007/s00520-026-10706-x

**Published:** 2026-04-30

**Authors:** Philip I. Chow, Lindley Slipetz, Katharine E. Daniel, Siqi Sun, Teague Henry

**Affiliations:** 1 Center for Behavioral Health & Technology, Department of Psychiatry and Neurobehavioral Sciences, University of Virginia School of Medicine, PO Box 801075, Charlottesville, VA USA; 2 Department of Psychology, University of Virginia, Charlottesville, VA USA

**Keywords:** Digital health, Cancer, Survivors, Mental health interventions

## Abstract

**Purpose:**

The growing population of cancer survivors in the US highlights the need for adaptive digital mental health treatments that can help address a large gap in mental health treatment. Although just-in-time adaptive interventions (JITAIs) hold promise for improving mental health outcomes, none have been developed specifically for cancer survivors, in part due to their complexity. The purpose of this study was to identify survivor-level factors that could inform the development and optimization of adaptive treatments aimed at improving affective outcomes in this population.

**Methods:**

A total of 426 adults diagnosed with cancer within the past five years participated in a 5-week observational study. Participants completed smartphone-based surveys three times per day assessing momentary affect, affective forecasting, emotion regulation attempts, social interaction quality, pain, and sleep duration from the previous night. Linear mixed-effects models were conducted at the momentary level to examine associations with positive affect (PA) and negative affect (NA).

**Results:**

Higher momentary PA was associated with longer sleep duration the previous night, lower pain, fewer emotion regulation attempts, higher-quality social interactions, and forecasting one’s future affect as more positive. In contrast, higher momentary NA was associated with shorter sleep duration the previous night, greater pain, more frequent emotion regulation attempts, poorer-quality social interactions, and forecasting one’s future affect as more negative.

**Conclusion:**

These findings identify several modifiable meta-emotion and psychosocial factors that may serve as promising targets for future JITAIs designed to improve affective well-being among cancer survivors.

**Supplementary Information:**

The online version contains supplementary material available at 10.1007/s00520-026-10706-x.

## Background

There were an estimated 18.6 million cancer survivors in the USA in 2025 [1], a number projected to exceed 22 million within the next decade [2]. As the number of survivors increases, so too does concern regarding the mental health burden faced by this population. Compared to the general population, cancer survivors are significantly more likely to experience mental health difficulties, particularly disturbances in mood such as depression and anxiety [3–5]. Despite this elevated risk, most survivors report unmet psychosocial needs and limited access to timely, tailored mental health support [6–8].

### Adaptive digital mental health treatments for cancer survivors

Digital mental health treatments (DMHTs) offer a promising approach to addressing this treatment gap. In particular, DMHTs that deliver empirically supportive interventions via smartphone apps or websites are easily accessible, cost-effective, time-efficient, and not restricted by the availability of local psychotherapists [9–11]. As DMHTs continue to harness emerging technologies (e.g., artificial intelligence) to offer new and innovative designs, features, and therapeutic strategies, there is untapped potential to develop DMHTs that are delivered in a personalized, just-in-time manner, known as just-in-time adaptive interventions (JITAIs [12]). JITAIs seek to provide the right kind of support, at the right time, based on dynamic information that is collected from individuals such as their mood or contextual states [12, 13]. Much of the progress in JITAIs has clustered around overt and readily sensed user behaviors, such as step count and diet, to trigger interventions [14].

Despite their potential for transforming mental health care, a recent review found that JITAIs for mental health are still in early stages of development, and the five publications reporting on existing JITAIs have core components that are only partly supported by empirical evidence [15]. One reason for these findings is due to the complexity of designing JITAIs for mental health constructs such as affect, depression, and anxiety; while no JITAIs have been developed to address mental health in cancer survivors, researchers seeking to do so must decide on a number of tailoring variables and decision rules to guide the intervention. The tailoring variable determines when and how an intervention is delivered, and is ideally based on a user’s behavior or experience such as a desire to regulate their emotions. Because it drives intervention delivery through the decision rules, the tailoring variable is a critical component of how a JITAI influences proximal outcomes such as momentary affect. The added complexity of developing JITAIs for mental health, however, requires a detailed understanding of both survivor-level characteristics and momentary processes that shape emotional experiences in daily life. However, to date, existing research on the psychosocial functioning of cancer survivors has largely relied on cross-sectional or sparsely sampled longitudinal designs, which overlooks the dynamic, within-person relationships between behavior, context, and affect that are most relevant for informing real-time intervention targets.

### Intensive longitudinal studies can inform JITAIs

Intensive longitudinal data is essential for understanding how emotion-related processes unfold in the daily lives of cancer survivors [16]. Although causal inferences are typically limited in observational studies because variables are not experimentally manipulated, these data are critical for advancing our understanding of survivors’ day-to-day experiences. Ecological momentary assessment (EMA), which involves repeatedly sampling individuals’ behaviors and experiences in real time [17, 18], typically via smartphones, provides a powerful framework for identifying proximal, modifiable factors in survivors, such as fluctuations in their pain, sleep, social interactions, and emotion regulation attempts, that could be targeted by JITAIs to improve their affect. Despite its promise and studies showing the feasibility of EMA in adults with cancer [19], the pool of available studies in adult cancer survivors remains limited in both sample size and scope [19, 20], constraining researchers’ ability to develop scalable, adaptive DMHTs. Therefore, the goal of the current study was to identify survivor-level factors associated with momentary affective experiences using intensive longitudinal EMA data captured via smartphones.

Positive and negative affect (PA and NA, respectively) are key outcomes for DMHTs targeting mental health in cancer survivors, as they both are barometers of well-being [21], are core components of mood and anxiety disorders [22], and shape future behavioral and cognitive trajectories [23, 24]. Researchers aiming to improve distal outcomes such as depression and anxiety symptom severity may therefore target proximal changes in daily life by reducing state NA and/or increasing state PA. Understanding the person-level factors that shape PA and NA can directly inform the timing and targeting of just-in-time adaptive interventions by identifying moments when individuals are most likely to benefit from support. Meta-emotion factors, which relate to how people think about, evaluate, or respond to their own emotions [25, 26], may serve as key targets for DMHTs seeking to modify affect in survivors. For example, frequent attempts to regulate emotions may not only signal persistent emotional distress or diminished PA [27, 28], they may also prolong NA if they are ineffective and contribute to a negative self-fulfilling prophecy. In addition, affective forecasting, or expectations about how one will feel in the future [29], is a cognitive-affective construct that has been linked to mood and anxiety disorders in the general population [30] yet has not been examined in cancer survivors. Among cancer survivors, elevated NA and/or diminished PA may bias expectations toward anticipating continued emotional distress, reinforcing maladaptive emotional cycles. By contrast, diminished NA and/or elevated PA may have the opposite effect of expecting to flourish emotionally, thereby breaking maladaptive emotional cycles.

In addition to meta-emotion factors, there are also several behavioral and contextual factors that likely play a role in shaping momentary affective experiences in survivors. Pain is commonly experienced by cancer survivors and has been robustly associated with negative emotional states [31], while poor social functioning is linked to increased loneliness and negative affect [32]. Sleep, a core biobehavioral process, has been shown to have a bidirectional relationship with affect [33, 34], such that poor sleep can worsen next-day mood, while heightened negative affect can disrupt subsequent sleep. Importantly, each of these factors is potentially observable and modifiable, making them strong candidates for targeting in JITAIs.

### The current study

The aim of the current study was to inform how to tailor adaptive DMHTs to cancer survivors' daily experiences, by identifying survivor-level meta-emotion and psychosocial factors associated with their PA and NA in daily life. Specifically, we examined pain, sleep duration, social interactions, affective forecasting, and emotion regulation attempts as candidate targets, testing their relationships with PA and NA. We hypothesized that higher pain, shorter sleep duration, poorer quality social interactions, and more unhelpful emotion regulation attempts would be associated with higher momentary NA and lower PA. We further hypothesized that current affect would be associated with expectations about future affect, consistent with affective forecasting literature in the general population. Finally, we explored whether survivor baseline characteristics (e.g., gender, race, cancer stage, and mental health history of depression and anxiety disorders) were associated with differences in affective states in daily life.

## Methods

### Participants

This decentralized study enrolled 426 adults in the US who were within five years of a cancer diagnosis (stages 0–4) and owned a smartphone. To minimize the risk of fraudulent responses, background checks were conducted on all pre-eligible participants. A total of 19 participants were excluded for the following reasons: 10 did not initiate EMA surveys, 9 requested to withdraw. The sample included some of the most common cancer types in the USA. Background characteristics of the total sample are presented in Table 1. This study was performed in line with the principles of the Declaration of Helsinki. Approval was granted by the Ethics Committee of the University of Virginia Institutional Review Board for Health Sciences Research (UVA IRB HSR#230,080).

**Table 1:** Sample demographics

Participant characteristics	*n* (%) or *M* (*SD*)
Age	48.73 (12.23)
Sex	
Male	37 (9.1%)
Female	367 (90.2%)
Other	3 (.7%)
Race	
White	351 (87%)
Black	20 (4.9%)
American Indian/Alaska Native	6 (1.5%)
Asian	17 (4.2%)
Native Hawaiian/Pacific Islander	1 (.3%)
Some other race	10 (2.5%)
Ethnicity	
Non-Hispanic	371 (93%)
Hispanic	30 (7.5%)
Type of cancer	
Breast	232 (57.3%)
Prostate	16 (4.0%)
Lung	13 (3.2%)
Colorectal	21 (5.2%)
Endometrial/ovarian/cervical	33 (8.2%)
Non-Hodgkin lymphoma	12 (3.0%)
Other*	78 (19.3%)
History of major depressive disorder	198 (49%)
History of anxiety disorder	214 (53%)

^*^Includes melanoma, bladder, kidney, pancreas, leukemia, thyroid, Hodgkin lymphoma, and brain cancers

### Procedure

Participants provided remote informed consent and were enrolled in a 5-week observational study. They completed a battery of online (REDCap) self-report questionnaires at baseline. Over the 5 weeks, participants received three surveys per day through the Effortless Assessment Research System (EARS) mobile application [35]. One survey was delivered randomly within each of the following time blocks: 8:00–10:00 AM, 1:00–3:00 PM, and 7:00–9:00 PM. Each survey could be completed within a two-hour window. Participants were compensated up to $100, with payment prorated based on EMA compliance. Only completed surveys were recorded.

### Measures

#### Demographics

Participants reported their age, gender, race, cancer diagnosis and stage, and whether they had previously received a diagnosis of depression or anxiety (Yes or No).

#### EMA

Participants were asked to rate the degree to which they felt happy, cheerful, pleased, sad, afraid, and miserable over the past hour using a 0 (not at all) to 10 (very much) scale for each emotion word. To increase internal validity, PA is the sum of the first three emotion words and NA is the sum of the last three emotion words. NA and PA ranged from 0 to 30, with higher values indicating more intense affect.^1^ Participants were also asked to rate, over the past hour, their (a) pain using a 0 (no pain) to 10 (worst pain imaginable) scale based on the pain intensity item from the PROMIS-29 Profile v2.0 [36]; (b) social interaction quality using a 0 (very poor) to 10 (very good) scale; (c) whether they had tried to change their feelings using a categorical scale of “no,” “yes and it helped,” “yes but it didn’t help,” and (d) sleep duration the previous night from 0–12 h (note, sleep duration was assessed in the morning EMAs only). Finally, to assess for affective forecasting, participants were asked to rate how they expected to feel in the next few hours using a −10 (very negative) to + 10 (very positive) scale. Table 2 contains means, standard deviations and intra-class correlations for the EMA variables.

**Table 2:** Means, standard deviations, and intraclass correlation coefficients for the EMA variables

	Mean	SD	ICC
PA	17.223	8.123	0.619
NA	3.825	5.48	0.596
Social interactions	6.383	2.738	0.602
Affect forecasting	4.815	3.788	0.665
Sleep	6.98	1.527	0.419
Pain	2.151	2.284	0.761

### Plan for analysis

Only EMA surveys that participants responded to were logged. Linear mixed-effects models were conducted separately for PA and NA at individual EMA timepoints.^2^ Numerical variables were standardized. Fixed effects included EMA-based predictors: pain, emotional regulation attempt, quality of interactions, forecast of mood, and sleep duration. Gender, age, depression diagnosis, anxiety diagnosis, cancer stage, and race (White/non-White) were included as covariates in the models. Each model also included lag-1 predictors. Specifically, all models included the previous timepoint (lag-1) PA and the previous timepoint NA. Outcome variables (and their lags) varied within day, while the previous night’s sleep duration was constant within day for a given participant.

Models were originally fit with maximal random effects, including random intercept and slopes for lag-1 PA, lag-1 NA, daily emotion regulation attempt, daily forecast of mood and daily sleep duration. Random slopes with near 0 variance were removed to improve convergence of final estimates. The PA model included a random intercept and random slopes for lag-1 PA, lag-1 NA, pain, social interactions, forecasting, and sleep quality. The NA model had the same random effects except for pain. Missing data was addressed using the multi-level multiple imputation strategy of Shafer and Yucel (2002), [37], as implemented in the *mitml* R package [38]. This approach uses mixed effects models to impute missing data, allowing for inter-individual variability to be properly accounted. We imputed missing values for PA, NA, daily interaction quality, affective forecasting, daily pain and daily sleep duration with mixed effects models containing fixed effects for gender, age and race, as well as random intercepts. Twenty datasets were imputed and estimated effects were combined as per Rubin (1987) [39]. All models were estimated using the *lmer* function from the lme4 package in R [40].

## Results

### EMA completion and descriptive statistics

On average, participants completed 55% of the EMA surveys, within the range reported by previous EMA studies in adults with cancer [19]. Among variables examined in prior studies of breast cancer survivors, the observed values were remarkably consistent. As seen in Table 2, participants reported much higher levels of PA than NA in daily life, which has been found in some prior EMA studies in breast cancer survivors [41, 42]. Their pain levels, while low on average, were also consistent with prior EMA research in breast cancer survivors that used a ten point scale [41]. Finally, breast cancer survivors in the current sample reported on average sleeping about 7 h per night which replicates findings in prior research [43].

### Baseline variables associated with PA

Results from mixed effects models for PA and NA can be found in Table 3. In terms of baseline characteristics and PA, having a prior depression diagnosis ($\beta =-.09,p=.03$) and having a more advanced stage of cancer was associated with lower PA at any given timepoint ($\beta$ = − 0.02, *p* = 0.03). Non-White individuals were more likely to report feeling more PA at any timepoint compared to White individuals ($\beta =.12,p=.02$).

**Table 3:** Fixed effect estimates for PA and NA outcome models. $\beta$ s reported are standardized effect sizes, with the exception of categorical variables (ER, gender, dxs, race) and cancer stage

	*PA*			*NA*		
	$\beta$	*t*	*p*	$\beta$	*t*	*P*
Previous timepoint PA	**.16**	**17.36**	** <.001**	**.02**	**2.37**	**.01**
Previous timepoint NA	−.01	− 1.04	.32	**.21**	**18.00**	** <.001**
Pain	−**.07**	− **9.54**	** <.001**	**.14**	**17.81**	** <.001**
Sleep	**.01**	**1.84**	**.04**	−**.02**	− **3.14**	**.006**
Emotion regulation- not helpful	−**.32**	− **12.8**	** <.001**	**.59**	**16.22**	** <.001**
Emotion regulation- helpful	−**.10**	− **6.31**	** <.001**	**.20**	**8.32**	** <.001**
Social interactions	**.35**	**60.11**	** <.001**	−**.11**	− **7.60**	** <.001**
Affect forecasting	**.29**	**28.48**	** <.001**	−**.25**	− **18.20**	** <.001**
Gender	.04	.31	.58	−.07	− 1.14	.25
Age	.02	1.11	.42	.02	1.09	.29
Prior depression diagnosis	−**.09**	− **1.32**	**.03**	−.05	− 1.24	.20
Prior anxiety diagnosis	−.04	− 1.27	.40	**.13**	**3.25**	**.001**
Cancer stage	−**.02**	− **2.49**	**.03**	−.01	−.70	.50
Race	**.12**	**2.57**	**.02**	.04	.68	.50

The pattern of results is remarkably similar if baseline levels of NA, PA, and time since diagnosis are added as covariates to the models

### EMA variables associated with PA

In terms of EMA variables, an increase in PA at the previous timepoint ($\beta$ = 0.16, *p* ≤ 0.001), but not NA at the previous timepoint, as well as reporting a higher quality of social interactions ($\beta =.35,p<.001$) and getting more sleep duration the previous night ($\beta =.01,p=.04$), were predictive of higher current PA. PA was also associated with affective forecasting, such that a higher level of current PA was associated with expecting to feel more positively in the near future $\left(\beta =.29,p<.001\right)$. By contrast, a higher level of pain ($\beta$ = − 0.07, *p* ≤ 0.001) and emotion regulation attempts, both helpful ($\beta$ = − 0.10, *p* < 0.001) and unhelpful ($\beta$ = − 0.32, *p* < 0.001) relative to no emotion regulation attempts, were associated with lower levels of current PA.

### Baseline variables associated with NA

In terms of baseline characteristics and NA, only having a past anxiety diagnosis was associated with a higher level of NA at any EMA timepoint ($\beta$ = 0.13, *p* = 0.001).

### EMA variables associated with PA

In terms of EMA variables, a high level of current NA was predicted by the previous timepoint NA ($\beta$ = 0.21, *p* ≤ 0.001) and to a lesser extent PA at the previous timepoint ($\beta$ = 0.02, *p* = 0.01), as well as getting less sleep the previous night ($\beta$ = − 0.02, *p* = 0.006). A higher level of pain ($\beta$ = 0.14, *p* ≤ 0.001) and frequency of emotion regulation attempts, both helpful ($\beta$ = 0.20, *p* < 0.001) and not helpful ($\beta$ = 0.59, *p* < 0.001), were all associated with a higher level of current NA. A higher level of NA was also related to expecting to feel more negatively in the future ($\beta$ = − 0.25, *p* ≤ 0.001). By contrast, lower current NA was associated with a higher quality of social interactions ($\beta$ = − 0.11, *p* ≤ 0.001).

## Discussion

Though it is important to not overinterpret these results given their correlational nature, results from this large observational study identify several promising person-level variables that may be of interest as tailoring variables for future JITAIs that aim to deliver support at moments of heightened need or opportunity to improve affect. In line with studies of inertia in affect [44], affect at the previous timepoint was a strong predictor of affect at any given timepoint, which magnifies the importance of helping survivors get on emotionally healthy trajectories. Findings also indicate that meta-emotion factors in cancer survivors, most notably emotion regulation attempts and affective forecasting, are closely tied to both their PA and NA in daily life.

With respect to emotion regulation, we found that attempts to regulate emotions were associated with lower momentary PA and higher momentary NA, regardless of whether those attempts were perceived as helpful. This pattern suggests that emotion regulation attempts may function less as indicators of successful coping and more as real-time markers of emotional distress. In other words, the act of trying to regulate one’s emotions may itself signal heightened emotional burden, independent of the effectiveness of the strategy employed. While some research in the general population has found that people tend to more frequently engage in emotion regulation when they are feeling negatively [27], to our knowledge this is the first time this finding has been reported in breast cancer survivors using EMA. This has direct implications for the design of JITAIs, as it highlights emotion regulation attempts as a potentially valuable tailoring variable for identifying moments of vulnerability and determining when intervention support may be most needed. Rather than waiting for affect to deteriorate further, JITAIs could treat regulation attempts as an early warning signal that an individual is struggling emotionally and may benefit from timely, targeted support. Moreover, these results suggest that interventions designed to help survivors better recognize when, where, and how to regulate their emotions, and to select strategies that are well matched to the emotional context, may improve affective outcomes by reducing the frequency of ineffective or unnecessary regulation attempts, consistent with emotion regulation theories emphasizing that perceived demands and emotional context are primary drivers of regulation efforts [27, 45]. For example, testing the comparative efficacy of various in-the-moment relaxation exercises, acceptance-based strategies, or cognitive restructuring techniques in a micro-randomized trial [46] may capitalize on survivors’ motivation to regulate while simultaneously guiding them toward evidence-based approaches [47]. Over time, such an intervention framework may help survivors become more skilled and efficient at self-regulating their affect, potentially reducing reliance on external prompts or supports.

Findings related to affective forecasting further highlight the central role of affect in shaping individuals’ expectations about their future and, by extension, their longer-term mental health trajectories. To our knowledge, this is the first EMA study to examine affective forecasting in cancer survivors. Higher momentary PA was associated with forecasting one’s future affect as more positive, consistent with the Broaden-and-Build theory [48], which posits that positive emotions expand attentional scope and cognitive flexibility and promote the accumulation of psychological and social resources over time. In contrast, higher momentary NA was associated with forecasting one’s future affect as more negative, aligning with cognitive-affective models suggesting that negative emotional states bias individuals toward more pessimistic expectations [49]. Together, these findings position affect as a critical proximal outcome for JITAIs, as momentary fluctuations in PA and NA may not only reflect current emotional states but also shape future-oriented beliefs that contribute to the development or maintenance of more distal outcomes, such as mood and anxiety disorders.

Beyond meta-emotion factors, consistent with the extant literature, our results also found that modifiable daily experiences, namely sleep, pain, and social interactions, are associated with PA and NA in cancer survivors. A unique contribution of our findings, however, relates to how sleep and affect are related in breast cancer survivors. Specifically, sleeping more was associated with higher next-day PA and lower levels of NA and worry, implicating sleep duration as an intervention target for promoting emotional resilience as well as downstream benefits for affective functioning. Consistent with some previous studies examining the association between pain and affect in adults with breast cancer [50, 51], our findings indicate that alleviating pain through efficacious behavioral interventions may improve emotional health, which may in turn influence future pain experiences, consistent with prior work showing bidirectional links between physical symptoms such as pain and affective states [52]. Finally, the association between higher-quality social interactions and improved affect suggests that enhancing social experiences may be another fruitful target for JITAIs. Interventions that promote behavioral activation, social engagement, or interpersonal effectiveness may help improve emotional well-being by increasing access to rewarding and supportive social experiences.

Our findings contribute to understanding of how demographic and cancer-related variables are associated with experiences of PA and NA in daily life, specifically highlighting the role of survivor characteristics of mental health history, cancer stage, and race. NA was significantly predicted by a prior anxiety diagnosis, whereas PA was significantly predicted by a prior depression diagnosis. This pattern does not imply, for example, that depression history is unrelated to NA; rather, conditional on other variables in the model, anxiety history contributed unique predictive information for NA, whereas depression history contributed unique information for PA. Taken together, these findings suggest that collecting more detailed information on mental health history, including trait-level vulnerability (e.g., trait anxiety), may improve the prediction of NA in future models. In addition, boosting PA may be a particularly important intervention goal for White survivors and those with more advanced-stage cancer, among whom lower PA was more pronounced. Tailoring intervention components to address these subgroup-specific vulnerabilities may help optimize the effectiveness of future JITAIs.

Finally, our findings raise important questions regarding the utility of targeting negative affect (NA) and positive affect (PA) as distinct intervention outcomes. The observed pattern of associations between NA and PA and the meta-emotion and psychosocial factors examined was largely overlapping, suggesting that interventions targeting these proximal factors may simultaneously reduce NA and enhance PA. Existing evidence suggests that NA and PA may be associated with different neurological pathways and outcomes [53, 54] and some evidence indicates that despite being correlated, NA and PA are not simply opposite ends of a single continuum and may not always co-occur [55, 56]. What remains relatively unknown is whether there is incremental benefit to independently targeting NA and PA within interventions, and whether doing so yields differential or additive effects on more distal clinical outcomes, such as the onset, severity, or persistence of mood and anxiety disorders. Future research that addresses this question empirically may help refine intervention targets and optimize the design of future digital and adaptive interventions by clarifying whether affective balance is best achieved through unified or differentiated treatment strategies.

### Limitations

The findings from this study should be considered in light of several limitations. The sample was relatively young compared to the broader population of cancer survivors, and the majority were female and diagnosed with breast cancer, which may limit generalizability to older adults, males, or survivors of other cancer types. Breast cancer survivors share a unique cluster of factors, including female sex [57, 58], high psychological burden [59, 60], and long-term physical symptoms from treatment such as fatigue, lymphedema, and weight gain [61, 62], that increases vulnerability to conditions such as insomnia and depression. However, these shared characteristics may not generalize to other survivor populations. Future studies using more heterogeneous samples should more closely examine the roles of sex and cancer treatment factors. Although we examined multiple models linking affect, meta-emotion factors, and psychosocial behaviors, the data are observational and correlational; therefore, causal inferences cannot be drawn. Future studies should isolate the specific pathways connecting pain, emotion regulation attempts, sleep, and social interactions to PA/NA, by manipulating one and seeing the effect on the other to determine their causal effects. While the overall EMA response rate was comparable to those reported in prior EMA studies including those among adults with cancer [19, 63], reasons for nonresponse were not assessed. The current study implemented a fairly intensive assessment strategy that may have been perceived as too burdensome for some participants. Future work may benefit from strategies to further improve adherence and to better understand barriers to responding, thereby strengthening reproducibility. Several of the EMA variables were assessed with custom items that have not been empirically validated. While the use of ultra-brief, single-item measures is common in EMA studies, it may also introduce psychometric limitations. Finally, although EMA allowed for rich repeated sampling of subjective affect, we did not include objective physiological or behavioral correlates (e.g., heart rate variability, skin conductance) that may provide complementary markers of affective states and further inform the development of JITAIs.

## Conclusion

This study advances our understanding of how meta-emotion processes, daily behaviors, and survivor characteristics are associated with momentary affect in the daily lives of adult cancer survivors. By identifying proximal, dynamic factors linked to PA and NA, these findings highlight the potential importance of sleep duration, pain, emotion regulation attempts, social interaction quality, and affective forecasting as potential targets and tailoring variables to increase PA and NA in future JITAIs. Continued efforts to disentangle these relationships and test them experimentally will be critical for developing timely, personalized behavioral interventions that effectively support emotional well-being in cancer survivorship.

## Supplementary Information

Below is the link to the electronic supplementary material.

ESM 1 (DOCX 22.4 KB)

## Data Availability

No datasets were generated or analysed during the current study.

## Footnotes

1 They were also asked the degree (0–10) to which they felt grateful, worried, and lonely using single item measures for each discrete type of affect, with higher values indicating more intense affect.

2 Additional results showing the relationship between the discrete emotions of gratitude, loneliness, and worry, can be found in the Supplemental Material. In general, there was considerable overlap in the patterns of associations between PA and gratitude, as well as between NA, worry, and loneliness, with some exceptions.
