# Supplementary material for: Identifying person-level factors to guide digital mental health treatments for cancer survivors: an ecological momentary assessment study
Source: Support Care Cancer. 2026 Apr 30;34(5):479. doi: 10.1007/s00520-026-10706-x (PMC13132892; doi:10.1007/s00520-026-10706-x)
Supplement: Supplementary file 1 — (DOCX 22.4 KB) [file 520_2026_10706_MOESM1_ESM.docx]

*Plan for Analysis of Worry, Gratitude, and Loneliness*

The plan for analysis was identical to what is described in the paper. Linear mixed-effects models were conducted separately for worry, gratitude, and loneliness at individual EMA timepoints. Numerical variables were standardized. Fixed effects included EMA-based predictors: pain, emotional regulation attempt, quality of interactions, forecast of mood, and sleep duration. Gender, age, depression diagnosis, anxiety diagnosis, cancer stage, and race (White/non-White) were included as covariates in the models. Each model also included lag-1 predictors. Specifically, all models included the previous timepoint (lag-1) PA and the previous timepoint NA; the worry model additionally included the previous timepoint worry, the loneliness model included the previous timepoint loneliness, and the gratitude model included the previous timepoint gratitude. Outcome variables (and their lags) varied within day, while the previous night’s sleep duration was constant within day for a given participant. The worry model included a random intercept and random slopes for lag-1 NA, lag-1 worry, forecasting, sleep duration, and emotional regulation attempt. The gratitude model included a random intercept and random slopes for lag-1 PA, lag-1 gratitude, pain, forecasting, sleep duration, and emotional regulation attempt. The loneliness model included a random intercept and random slopes for lag-1 NA, lag-1 loneliness, interaction quality, forecasting and emotional regulation attempt. Similar to the primary analyses, we imputed missing values for gratitude, worry, and loneliness. Descriptive statistics for these variables can be found in Table 1S.

*Results*

Results from mixed effects models can be found in Table 2S.

Gratitude*.* Higher gratitude at any timepoint was predicted by the previous timepoint PA ($\beta$ = 0.03, *p* = .004) and gratitude ($\beta$ = 0.13, *p* = <.001). Similar to findings for PA, a higher quality of social interactions at a given timepoint was associated with a higher level of gratitude ($\beta$ = 0.25, *p* = <.001), and feeling more grateful was associated with expecting to feel more positively in the future ($\beta$ = 0.20, *p* = <.001). In terms of emotion regulation attempts, only more frequent unhelpful attempts were associated with lower levels of gratitude ($\beta$ =- 0.19, *p* = <.001) relative to no emotion regulation attempt. A higher level of pain was associated with a lower level of momentary gratitude ($\beta$ = -0.05, *p* = <.001). In terms of baseline characteristics, age was significantly associated with gratitude, such that older adults reported feeling more grateful at any given timepoint compared to younger adults ($\beta$ = 0.10, *p* = <.001). Moreover, race was also associated with gratitude, such that non-White individuals were more likely to report feeling more grateful at any timepoint compared to White individuals ($\beta$ = 0.23, *p* = .002).

Worry. Momentary worry was significantly predicted by the previous timepoint NA (β= .06, p< .001), PA (β= .03,p= .001), and worry (β= .18,p< .001), , and lower momentary worry was associated with an increase in sleep duration the previous night (β = -.02, p =.007). Similar to findings for NA, a higher level of worry was associated with a higher level of pain (β = 0.09, p = <.001), frequency of emotion regulation attempts, both helpful (β = .28, p <.001) and unhelpful (β = 0.48, p <.001) relative to no emotion regulation attempt, and past anxiety diagnosis (β = .14, p =.004). By contrast, an increase in quality of social interactions (β = -.08, p <.001) was associated with lower momentary worry. A higher level of worry was also associated with expecting to feel more negatively in the future (β = -.21, p <.001).

Loneliness. Momentary loneliness was predicted by the previous timepoint NA (β = .04, p <.001) and loneliness (β = .18, p <.001). Similar to findings for NA and worry, a higher level of momentary loneliness was associated with a higher level of pain (β = .06, p <.001), helpful (β = .12, p <.001) and unhelpful (β = .37, p <.001) emotion regulation attempts relative to no emotion regulation attempt. A decrease in loneliness was predicted by an increase in quality of social interactions (β = -.19, p <.001) and more positive forecasted affect (β = -.09, p <.001). In terms of baseline characteristics, a higher level of momentary loneliness was predicted by older age (β = .05, p =.01) and having a past anxiety diagnosis (β = .09, p =.02).

*Discussion*

Overall, there was considerable overlap in the patterns of associations between PA and gratitude, as well as between NA, worry, and loneliness. Collectively, the findings suggest that assessing discrete emotions may be redundant with PA or NA in many contexts, with some exceptions. Specifically, helpful emotion regulation attempts, sleep duration the previous night, and having a past depression diagnosis were associated with momentary positive affect but not momentary gratitude. Having a more advanced cancer stage was also predictive of lower momentary PA but not gratitude, suggesting that an advanced stage of disease may have a broader dampening effect on mood. By contrast, older age was linked to higher momentary gratitude specifically, consistent with research showing increases in conscientiousness and emotional stability with age. Yet, being older was also associated with higher momentary loneliness specifically among negative affective states. These results highlight a potential tension between higher momentary gratitude and rising loneliness among older adults that may be influenced by the level of social support they receive. Future studies should identify potential mechanisms linking gratitude and loneliness in older adults, as well as moderators that differentiate between older adults who have high gratitude/low loneliness and those with low gratitude/high loneliness. Taken together, these findings suggest that specific affective states may be more or less relevant depending on the health outcome of interest. Discrete affective states were most strongly predicted by their own prior timepoint values (i.e., lag-1 predictors), rather than general PA or NA. Collectively, these findings provide some support for the potential usefulness of assessing for discrete emotional states depending on the outcome of interest, consistent with research demonstrating the predictive utility of discrete emotions in psychopathology.

**Table 1S.** Means, Standard Deviations, and Intraclass Correlation Coefficients for the EMA variables of Worry, Gratitude, and Loneliness

|  | Mean | SD | ICC |
| --- | --- | --- | --- |
| Worry | 2.288 | 2.636 | 0.546 |
| Gratitude | 6.237 | 3.043 | 0.633 |
| Loneliness | 1.618 | 2.485 | 0.618 |

**Table 2S.** Fixed effect estimates for Worry, Loneliness, and Gratitude, outcome models. $\beta$s reported are standardized effect sizes, with the exception of categorical variables (ER, Gender, dxs, Race) and Cancer Stage.

|  | *Worry* | | | *Loneliness* | | | *Gratitude* | | |
| --- | --- | --- | --- | --- | --- | --- | --- | --- | --- |
|  | $\beta$ | *t* | *p* | $\beta$ | *t* | *P* | $\beta$ | *t* | *P* |
| Previous PA | **.03** | **3.77** | **.001** | .01 | 1.80 | .37 | **.03** | **2.71** | **.004** |
| Previous NA | **.06** | **5.47** | **<.001** | **.04** | **4.30** | **<.001** | .01 | 1.52 | .14 |
| Previous Worry | **.18** | **16.14** | **<.001** |  |  |  |  |  |  |
| Previous Lonely |  |  |  | **.18** | **15.06** | **<.001** |  |  |  |
| Previous Gratitude |  |  |  |  |  |  | **.13** | **11.73** | **<.001** |
| Pain | **.09** | **10.15** | **<.001** | **.06** | **8.55** | **<.001** | **-.05** | **-6.18** | **<.001** |
| Sleep | **-.02** | **-2.70** | **.01** | .002 | .43 | .69 | -.007 | -1.12 | .27 |
| ER- not helpful | **.48** | **14.27** | **<.001** | **.37** | **11.36** | **<.001** | **-.19** | **-7.92** | **<.001** |
| ER- helpful | **.28** | **11.66** | **<.001** | **.12** | **5.91** | **<.001** | .01 | .65 | .52 |
| Social Interactions | **-.08** | **-12.05** | **<.001** | **-.19** | **-29.40** | **<.001** | **.25** | **42.95** | **<.001** |
| Affect Forecasting | **-.21** | **-27.39** | **<.001** | **-.09** | **-15.27** | **<.001** | **.20** | **29.80** | **<.001** |
| Gender | .08 | 1.41 | .29 | -.08 | -.98 | .20 | .05 | .19 | .58 |
| Age | .01 | .23 | .52 | **.05** | **1.60** | **.01** | **.100** | **3.94** | **<.001** |
| Past Depression dx | -.01 | -.03 | .81 | .02 | 1.10 | .69 | -.08 | -1.51 | .17 |
| Past Anxiety dx | **.14** | **2.23** | **.004** | **.09** | **2.67** | **.02** | .04 | .81 | .45 |
| Cancer Stage | -.02 | -1.03 | .15 | -.01 | -1.70 | .12 | -.01 | -1.07 | .34 |
| Race | .10 | 1.44 | .12 | .04 | .14 | .44 | **.23** | **3.21** | **.002** |
